# Microhabitat partitioning between sympatric intertidal fish species highlights the importance of sediment composition in gravel beach conservation

**DOI:** 10.1002/ece3.10302

**Published:** 2023-07-10

**Authors:** Maximilian Wagner, Čedomir Benac, Maja Pamić, Sandra Bračun, Martin Ladner, Pia Clarissa Plakolm, Stephan Koblmüller, Hannes Svardal, Simon J. Brandl

**Affiliations:** ^1^ Institute of Biology University of Graz Graz Austria; ^2^ Department of Biology University of Antwerp Antwerp Belgium; ^3^ Faculty of Civil Engineering University of Rijeka Rijeka Croatia; ^4^ Faculty of Science University of Zagreb Zagreb Croatia; ^5^ Public Institution Kamenjak Premantura Croatia; ^6^ Department of Marine Science, Marine Science Institute The University of Texas at Austin Port Aransas Texas USA

**Keywords:** Adriatic Sea, Croatia, cryptobenthic fish, divergent ecological selection, ecological diversification, Mediterranean Sea, tourism

## Abstract

Gravel beaches in the Mediterranean ecoregion represent an economically important and unique habitat type. Yet, burgeoning tourism, intensive coastal development and artificial nourishment of beaches may jeopardize their ecological communities. To date, species that reside on gravel beaches and the consequences of beach alterations are poorly understood, which hampers the development of a sustainable coastal tourism industry along the region's shorelines. Using a simple collection method based on dredging buckets through the intertidal section of beaches, we quantified the microhabitat association of two sympatric clingfish species in the genus *Gouania* at seven natural and an artificial gravel beach based on sediment characteristics. We hypothesized that slender (*G. pigra*) and stout (*G. adriatica*) morphotypes would partition interstitial niche space based on sediment size, which may affect the vulnerability of the species to changes in gravel beach composition due to coastal development. We detected substantial differences in gravel composition within and among the sampled beaches which suggests scope for microhabitat partitioning in *Gouania*. Indeed, we found significant relationships between species identity and the presence/absence and abundance of individuals in hauls based on their positioning on PC1. Our results suggest that modifications of gravel beaches through coastal development, including beach nourishment, intensifying coastal erosion, or artificial beach creation, may have detrimental consequences for the two species if sediment types or sizes are altered. We posit that, given the simplicity and efficacy of our sampling method and the sensitivity of *Gouania* species to prevailing gravel composition, the genus could serve as an important indicator for gravel beach management in the Mediterranean ecoregion.

## Graphical abstract

We investigated the microhabitat association of two sympatric occurring species of the clingfish Gouania in the northern Adriatic Sea. The two species show diverging patterns of microhabitat use which suggest local niche partitioning as a driver in the diversification process of the genus.

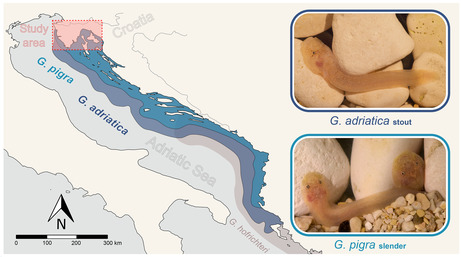

## 1 INTRODUCTION

Across the globe, coastal ecosystems are threatened by anthropogenic activities such as habitat alteration, pollution, overexploitation, rising sea levels and global warming (Costello et al., 2010; He & Silliman, 2019; Pikelj & Juračić, 2013; Ramesh et al., 2015). While many of these threats unfold at large spatial scales (Cramer et al., 2018; Giorgi, 2006), local coastal development can greatly affect the geological, oceanographic and ecological dynamics of near shore environments (Bulleri & Chapman, 2010; Burt & Bartholomew, 2019; Drius et al., 2019; Pikelj & Juračić, 2013). For instance, coastal restructuring negatively impacts important ecosystems such as sandy dunes, seagrass meadows, mangrove forests or biogenic reefs, which provide physical protection to shorelines and harbour diverse near‐shore biological communities (Burt & Bartholomew, 2019; Heard et al., 2021; Martínez et al., 2008; Pruckner et al., 2022). Nonetheless, especially in densely populated areas with extensive tourism infrastructure, the development and management of coastal ecosystems have become inevitable to create additional urban living space or mitigate the effects of coastal erosion and flooding (Ramesh et al., 2015; Staudt et al., 2021; Temmerman et al., 2013).

**FIGURE 1:**
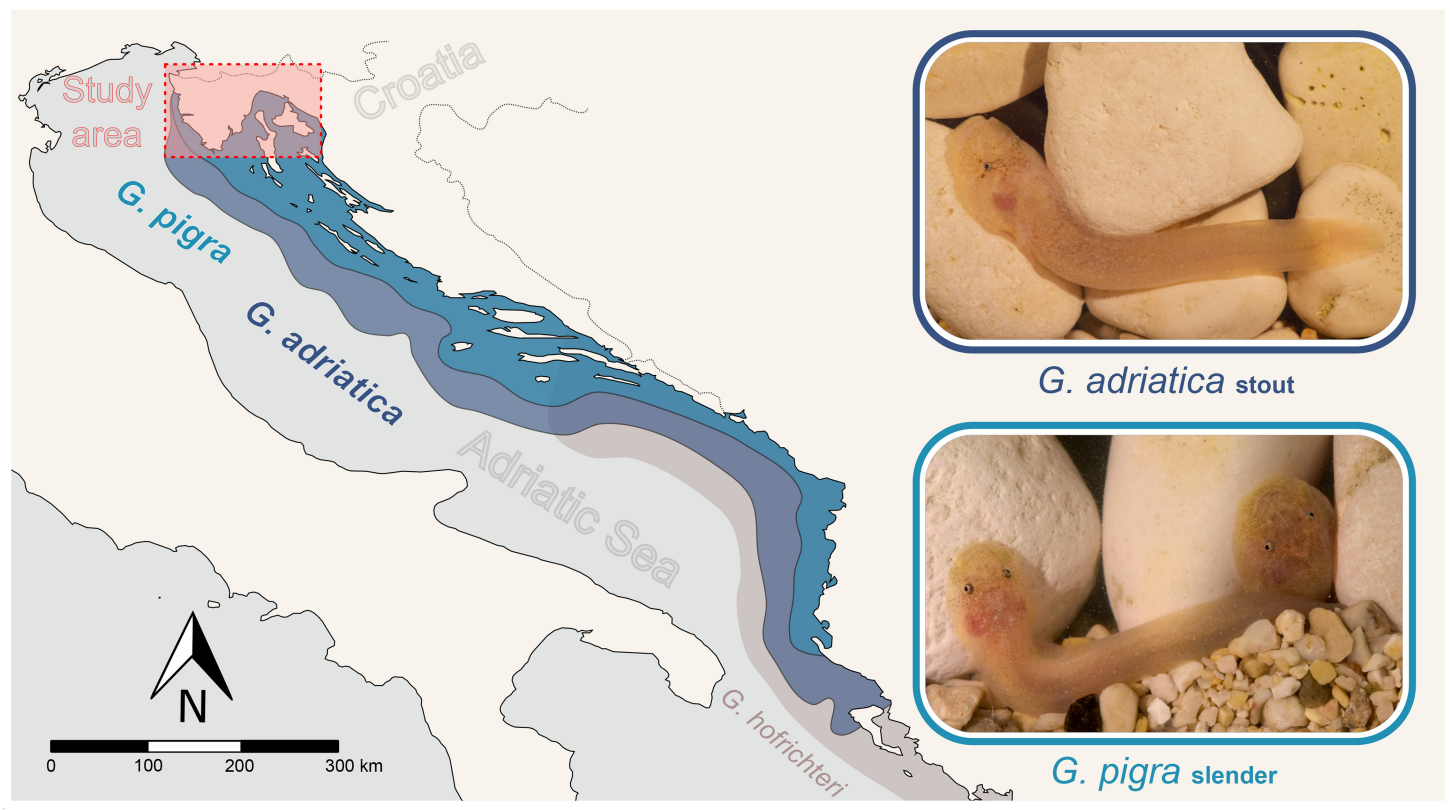
Map of the Adriatic Sea, including the study area (red rectangle), the studied *Gouania* species, their distribution ranges (light and dark blue shading). Additionally, the potential distribution range of *G. hofrichteri* is shown in light grey (with a northernmost record from Pelješac peninsula).

The Mediterranean ecoregion is one of the first and most strongly anthropogenically impacted areas worldwide. While early human activities mainly shaped terrestrial landscapes, the development of coastal infrastructure has become increasingly necessary to cope with increasing demands of tourism and industry (e.g., Carević, 2020; Carević et al., 2022; Pikelj, Ružić, Ilić, et al., 2018). This is especially true for the eastern Adriatic coast of Croatia, which has become one of Europe's leading summer tourist destinations in recent years (Orsini & Ostojić, 2018). Beaches along the Croatian coastline are typically formed by flysch rock assemblages or carbonate gravel pockets but account for only 5 per cent of the total length of the entire eastern Adriatic coastline, which is dominated by rocky outcrops and cliffs (Pikelj & Juračić, 2013). Hence, beaches represent a relatively rare and highly fragmented habitat type in the eastern Adriatic. They are maintained by a complex equilibrium of geological, biological, and oceanographic forces. However, these dynamics are increasingly disturbed in areas of intensifying urbanization and structural alterations of the natural coastline (Benac et al., 2021; Pikelj, Ružić, Ilić, et al., 2018). Additionally, rising sea levels, storms and extreme tidal events are expected to enhance coastal erosion in the Adriatic region, further affecting these ecosystems (Bonaldo et al., 2017; Gallina et al., 2019; Orlić & Pasarić, 2013; Ružić et al., 2014, 2021; Torresan et al., 2012, 2019; Tsimplis et al., 2012). To mitigate the effects of natural or anthropogenic degradation of beach areas and to enhance beach availability for tourism, common management strategies include restoration through artificial nourishment of beaches (Carević, 2020; Pikelj & Juračić, 2013) and the creation of new artificial beach areas (Carević, 2020; Carević et al., 2022; Pikelj & Juračić, 2013; Figure 1). Consequently, between 2017 and 2018, the total amount of artificially deposited material for beach creation has almost quadrupled from 21 to 80 tons (Carević et al., 2022). While these actions bring short‐term economic benefits, they may adversely affect ecological dynamics in gravel beach ecosystems, particularly if the wrong type or quantity of sediment is applied, such as waste material from quarries (Carević, 2020; Carević et al., 2022; Colosio et al., 2007; Drius et al., 2019; Parkinson & Ogurcak, 2018; Peterson & Bishop, 2005; Speybroeck et al., 2006; Staudt et al., 2021). Understanding the effects of beach alterations on the biota that reside in natural and artificial gravel beaches in Croatia will be crucial for developing a sustainable coastal tourism industry along the region's shoreline.

From a biological perspective, marine gravel beaches are one of the most demanding ecosystems on Earth. Specifically, heavy mechanical disturbance and wave action, as well as constant changes in abiotic and biotic conditions are only tolerated by few species with particular adaptations (Ronowicz, 2005). Among marine vertebrates, only two lineages of cryptobenthic fishes – the clingfish genus *Gouania* (Gobiesocidae) and the gobiid genus *Luciogobius* (Gobiidae) – are known to have successfully colonized the interstitial spaces of intertidal gravel beaches (Wagner et al., 2019; Yamada et al., 2009). Notably, both genera have converged on the same morphological adaptations (e.g., extensive vertebral segmentation, elongated, scale‐less bodies, reduced fins), which indicates strong selective pressure induced by the prevailing habitat conditions (Wagner et al., 2019, 2021; Yamada et al., 2009). The cryptobenthic clingfish genus *Gouania* is a Mediterranean endemic, which currently includes five species that primarily separate into two major phenotypes along an axis of body elongation (Wagner et al., 2019, 2021). Slender *Gouania* (as opposed to their stout counterparts) are characterized by a larger number of vertebrae and smaller eyes, which may suggest differences in the use of interstitial microhabitats as an increased number of vertebrae can result in higher body flexibility to permit locomotion in narrower spaces (Costa et al., 2020; Wagner et al., 2019, 2021; Yamada et al., 2009). Ecological diversification, resulting from niche partitioning among closely related species, is common in cryptobenthic fish taxa (Ahmadia et al., 2018; Brandl et al., 2020, 2022; Dirnwöber & Herler, 2007; Goatley et al., 2016; Herler, 2007; Kovačić et al., 2012; Rüber et al., 2003; Tornabene et al., 2013; Yamada et al., 2009), and compared to larger fishes, the small body size of many cryptobenthics has permitted partitioning of food resources and microhabitats at a particularly granular scale (Brandl et al., 2018, 2020, 2022; Longenecker, 2007). Therefore, sympatric *Gouania* species with different morphologies may coexist stably by partitioning the microhabitats and resources available on gravel beaches. However, species coexistence might be disturbed if the composition of gravel beaches is altered. This, in turn, may have strong consequences on the structure and function of interstitial communities on gravel beaches, especially given that *Gouania* are likely to function as apex predators in these communities (Hofrichter, 1995).

In the present study, we used a new ecological survey technique to investigate the potential vulnerability of sympatric *Gouania* species to coastal development and anthropogenic modification of gravel beaches in the Adriatic. Thus far, the only ecological study available on *Gouania* (Hofrichter & Patzner, 2000) was conducted prior to the major taxonomic revision of the genus (Wagner et al., 2021), therefore neglecting potential interspecific and local variation. Specifically, we used a simple hand‐held dredging method to assess the microhabitat association of two divergent phenotypes from the Adriatic Sea, stout *G. adriatica* and slender *G. pigra*, to characterize potential niche partitioning among these closely related species and its possible consequences for the species' vulnerabilities to coastal development. We compared sediment composition of eight different gravel beaches from the northern part of the Adriatic (Istria and Kvarner area) and found subtle but robust correlations between granulometric composition and *Gouania* populations, suggesting that changing gravel composition may affect population dynamics of the two species. We discuss our findings in the light of ongoing as well as future beach nourishment activities and the crucial role *Gouania* may play for the design and monitoring of artificial gravel beaches in the context of habitat preservation and restoration.

## 2 METHODS

### 2.1 Sampling and geological characterization of the beaches

**FIGURE 2:**
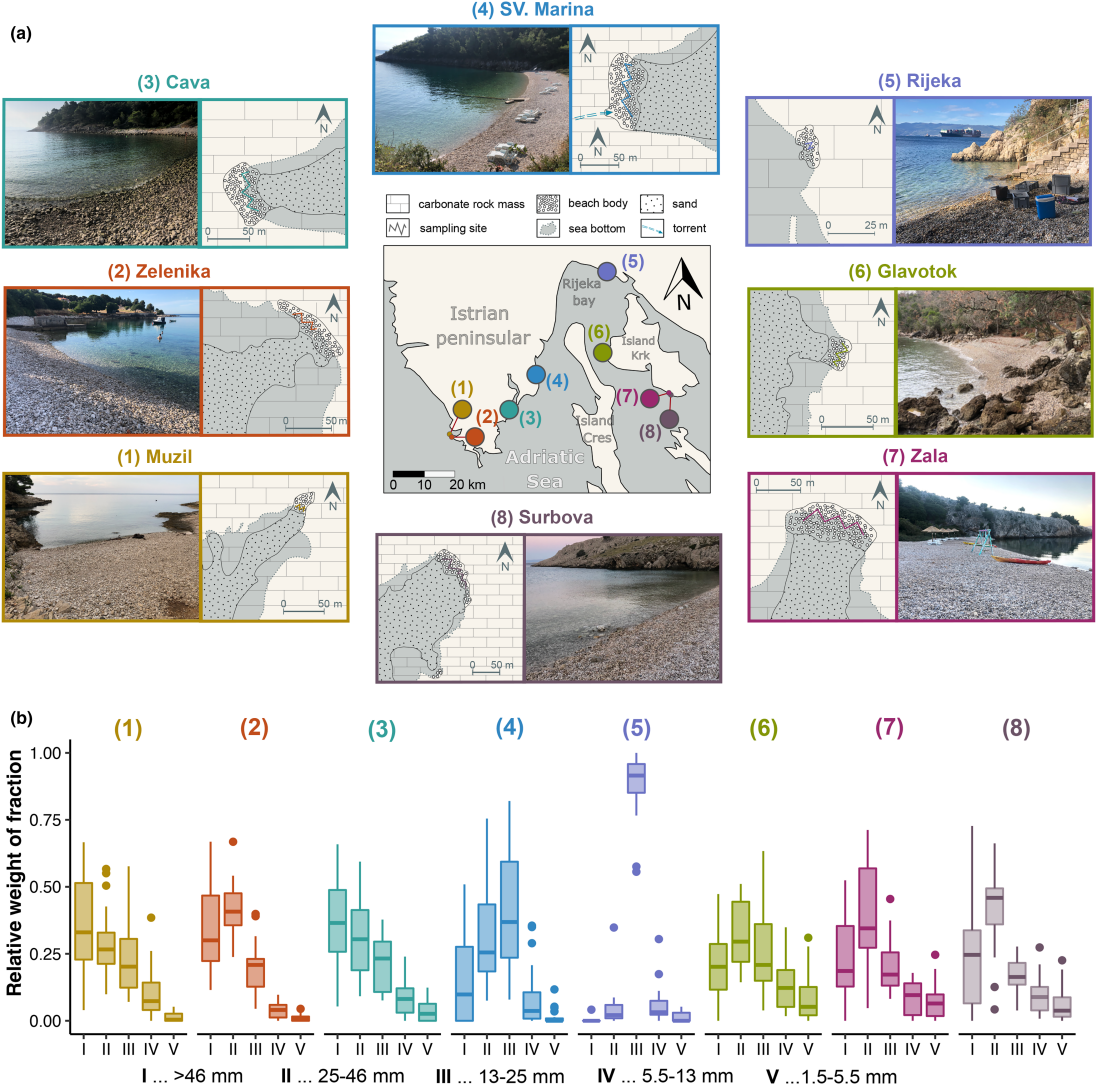
Geological fabric and sedimentologic composition of the eight investigated sites from the northern Adriatic Sea (Istria and Kvarner area). (a) Representative images and locations of the study sites. (b) Relative weight distributions of five investigated size fractions (I: >46 mm, II: 25–46 mm, III: 13–25 mm, IV: 5.5–13 mm, V: 1.5–5.5 mm) from 20 random hauls per site.

In total, we assessed the gravel composition of eight beaches in the Northern Adriatic Sea (Kvarner area, and Istrian peninsular) from July to October 2021 (Figures 1 and 2a).

We initially examined the beaches in the study area to determine the presence of *Gouania* for inclusion in the study. Subsequently, we selected for practical reasons, such as accessibility and minimal disturbance to the public. We collected sediment and its associated fish community by dredging a customized iron bucked with a volume of 10 L through the gravel grains down to a water depth of 1 m. At each site (i.e., beach), we performed 20 hauls haphazardly distributed across the beach area that encompassed water depths and habitat typically inhabited by *Gouania* (see Table 1), yielding a total of 160 hauls. All hauls were taken by the same researcher (M. Wagner) to ensure consistency in the sampling procedure. We categorically assigned the depth of each haul as either (1) surface, (2) shallow (a depth where sampling was possible without full submersion of the surveyor) and (3) deep (a depth where sampling required full submersion of the surveyor).

**TABLE 1:** Overview of the investigated beaches and sampling sites.

Beach	(1) Muzil	(2) Zelenika	(3) Cava	(4) Sv. Marina	(5) Rijeka	(6) Glavotok	(7) Zala	(8) Surbova
Location	Muzilj cove	Stoja cove	Kaval cove	Guboka cove	Rijeka Pećine	Komoštrin cove	Zala cove	Surbova cove
Sampling dates	29.09.21, 19.10.21	19.10.20–25.10.20, 02.07.21, 03.07.21, 09.07.21	28.07.21, 29.07.21	25.09.21	15.09.21, 13.10.21	02.09.21	14.09.21	13.09.21
Coordinates	44.861285, 13.806681	44.859607, 13.821344	44.933708, 14.041509	45.028383, 14.154949	45.314558, 14.469822	45.083657, 14.432419	44.948827, 14.699382	44.945866, 14.706058
Length (m)	20	80^a^	70	90	20	30	100	70
Width (m) (below M.S.L.)	25 (10)	15–20 (10–15)	30 (15)	30 (17)	13 (10)	10 (5)	30–40 (20–30)	25–35 (15–25)
Orientation	SW	SSW	NE	E	SW	WNW	SSW	SW
Beach origin	Long‐term marine erosion	Long‐term marine erosion	Long‐term marine erosion	Long‐term marine erosion	Allochthonous sediment	Long‐term marine erosion	Long‐term marine erosion	Long‐term marine erosion
Pebble shape	Rounded to sub‐rounded, equant, bladed	Rounded to sub‐rounded, equant, discoidal	Rounded to sub‐rounded, equant, spherical	Rounded to sub‐rounded, spherical, equant, discoidal	Rounded to sub‐rounded, spherical, equant, discoidal	Rounded to sub‐rounded, equant, spherical	Rounded to sub‐rounded, spherical, equant, discoidal	Rounded to sub‐rounded, spherical, equant, discoidal
Mean (kg) per haul (SD)	12.71 (1.93)	11.88 (2.26)	13.08 (1.57)	13.62 (1.83)	11.19 (2.31)	12.00 (2.04)	13.32 (1.30)	12.76 (2.57)

*Note*: In total we investigated 8 different locations, whereas their geographical location in the Kvarner area can be found in Figure 2. For each location we deliver the sampling dates, coordinates length, width, orientation, the origin and shape the sediments, as well as the mean sediment amount in kg (plus standard deviation).

Abbreviation: M.S.L., Mean Sea Level.

^a^ Samples were taken from only a ca. 40 m stretch just north of the concrete jetty (see Figure 2).

After each haul, we separated the fish from the sediment on a plastic tray, anesthetized the collected fish with MS‐222, placed them in an ice‐water slurry and took standardized photographs for morphometric measurements. To investigate morphological relationships, we measured the standard length (SL), total length (TL), head width at posterior head invagination (HW), head depth at anterior sucking disc edge (HD) and preorbital length from the anterior tip of the eye to the tip of snout (preOrb) of each collected fish. We also separated the sediment from each haul in the field using a custom‐made sieve apparatus that divided sediment grains into the five fractions >46, 25–46, 13–25, 5.5–13 and 1.5–13 mm, roughly representing the size spectrum of sediment grains that represent typical *Gouania* habitat. After separating the fractions, we weighed and calculated the total mass of each fraction (i.e., the difference of the full weight including sediment minus the empty weight of the apparatus). We additionally randomly collected sediment from each site to investigate grain size, roundness and sphericity as well as the shape of grains from each site following standard sedimentological procedures (Coe, 2010).

### 2.2 Statistical analyses

From the total weight of each fraction, we calculated the relative proportion of fractions in each haul. We also calculated the estimated mean grain size (in mm) and sorting index of each haul according to Folk and Ward (1957) using the geometric values in GRADISTAT v.8.0 (Blott & Pye, 2001). All other statistical analyses were conducted in R v. 4.0 (R Core Team, 2021).

We conducted a Principal Components Analysis (PCA) on the relative weight data of sediments across fractions in each haul. To test whether the relative sediment compositions of each haul correlated with species' abundances, locations, or their interaction, we conducted a permutational multivariate analysis of variance (PERMANOVA), where species * location was used as the explanatory variable. We also performed a one‐way analysis of variance (ANOVA) to compare the two granulometric parameters (sorting and estimated mean gravel size) across different locations.

We then used the scores of each sample on PC1 and PC2 (which together explained 90.5% of the variability in the data) to further examine microhabitat associations in the two species. Specifically, we used a logistic regression to assess the relationship between the PC1 score of a sediment sample and the presence (1) or absence (0) of the two species. Additionally, we investigated the abundance of each species in relation to the first PC axis using a zero‐inflated Poisson model and assessed confidence intervals using a bootstrapping procedure with 10,000 bootstraps.

Finally, to infer temporal changes of sediment composition and species microhabitat associations we also compared data obtained from this study with a pilot study, conducted in October 2020, from the site Zelenika. In the pilot study, only hauls that contained fishes were considered; therefore, we subset the 2021 data to cases where either of the two species or both were found in a given haul. We then again conducted a PCA on the relative sediment composition of hauls from both years. To assess changes in body size during the sampling period, which could be an indicator for ontogenetic habitat shifts, we compared the TL of all fishes collected across both years and species. If more than one individual of each species was caught in the same haul, we calculated the mean TL for the haul. Finally, we assessed linear relationships between habitat parameters (PC1 and PC2) and TL.

## 3 RESULTS

In total, we obtained and investigated 2011.28 kg of sediment from four sites on the Istrian peninsula, one at the northern tip of the Rijeka Bay (Rijeka) and from three sites on the island of Krk (Figure 2a, Table 1). On average, each haul yielded 12.57 kg (±2.11 kg SD) of sediment. The orientation of the beaches differed, with five facing southwards (from SW to SSW), one eastward, one north‐eastward and one westward (WNW). Shapes of the gravel sediment were broadly comparable, from rounded to sub‐rounded as well as equant and sometimes bladed, discoidal and spherical. All investigated beaches were formed through long‐term marine erosion of (dolomitic) limestones, and carbonate breccias of different lithogenic origin, and local transportation by torrential flows. Thus, the gravel on most beaches was of natural origin, which is also reflected in the geological makeup of the investigated coves. However, the geological map at the beach in Rijeka clearly indicates a secondary artificial allochthonous gravel nourishment of this site. The beaches differed in length and width ranging from 20–100 m to 10–40 m, respectively. We observed an overall variation in the distribution of relative sediment fractions at different locations (Figure 2b). A more detailed geological description and characterization for each beach can be found in Appendix S1.

**FIGURE 3:**
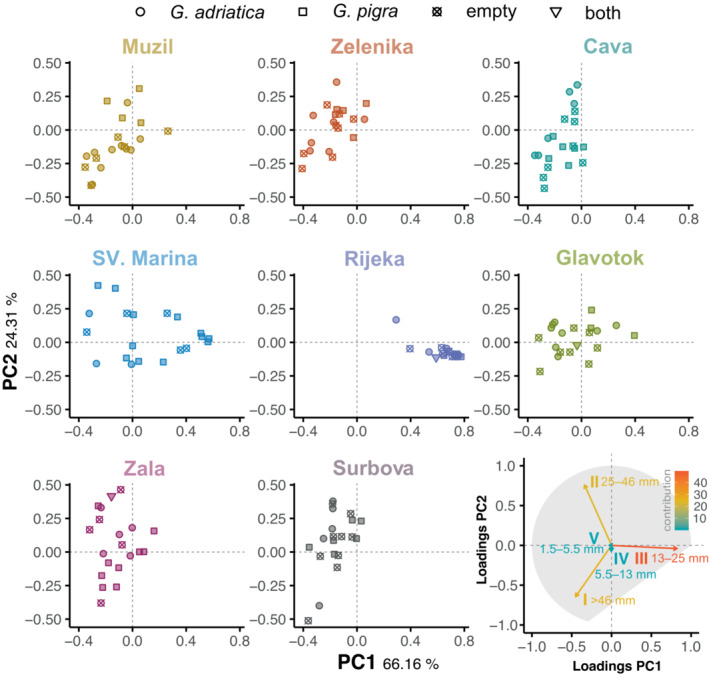
Principal components analysis (PCA) based on the relative weights of five size fractions (I: >46 mm, II: 25–46 mm, III: 13–25 mm, IV: 5.5–13 mm, V: 1.5–5.5 mm) separately shown for each site. The shape of data points represents the haul category (*Gouania adriatica*, *G*. *pigra*, empty haul or both species in the same haul). Percentages on the *x*‐ and *y*‐axis labels indicate explained variation. Loadings for the first two PC axis and their relative contributions are shown in the bottom right. For a better readability of the graph the single locations are plotted in different panels, even though they come from the same PCA.

The Principal Component Analysis (PCA) of the relative sediment fractions (Figure 3) showed some separation among sites, but also substantial heterogeneity among hauls within each beach. PC1 and PC2 explained 66.16% and 23.31% of the variation in the dataset, respectively. The distribution of hauls on PC1 corresponds with an increase towards the fraction III (13–25 mm), while fractions I (>46 mm) and II (25–46 mm) mainly contribute to changes along the second PC axis, with increasing values of PC2 leading to lower values of fraction I and higher values of fraction II (Figure 3; Appendix S2). The three locations on the southern Istrian peninsula (Muzil, Zelenika, Cava) as well as the sampling sites on the southern parts of Krk (Zala, Surbova) clustered towards the negative side of PC1, but varied widely along PC2. In contrast, Sv. Marina and Glavotok varied more strongly along PC1 and showed less variation on PC2. Lowest overall dispersion in multivariate space was found in Rijeka. Furthermore, we found significant differences in the sorting index between the locations (Appendix S3; ANOVA, *p* = 5.2e−7), but not for the estimated mean gravel size (Appendix S3; ANOVA, *p* = .2). The three southern Istrian sampling sites (Muzil, Zelenika and Cava) displayed lower sorting index values than the other sites (Appendix S3). Overall, we found a significant effect of location on sediment fractions, which explained 50% of the total variation in the PERMANOVA (*p* = .001; Appendix S4).

Of the 160 hauls, 102 contained individuals of one of the two sympatric *Gouania* species (50 *G. adriatica* and 52 *G. pigra*), 55 were empty and only 3 hauls yielded both species (Appendix S5). We collected a total of 73 and 83 individuals of *G. adriatica* (stout morphotype) and *G. pigra* (slender morphotype), respectively. *Gouania adriatica* was more abundant at Glavotok and Muzil, and *G. pigra* clearly dominated the beaches Zala and Sv. Marina (Appendix S5). Additionally, *G. adriatica* was never found at the waterline, but increased in numbers with depth (Appendix S5). We found no differences in the overall body size (TL and SL) between the two species, but lower relative values for the three morphometric measurements (HW, HD and preOrb) for *G. pigra* (Appendix S6).

**FIGURE 4:**
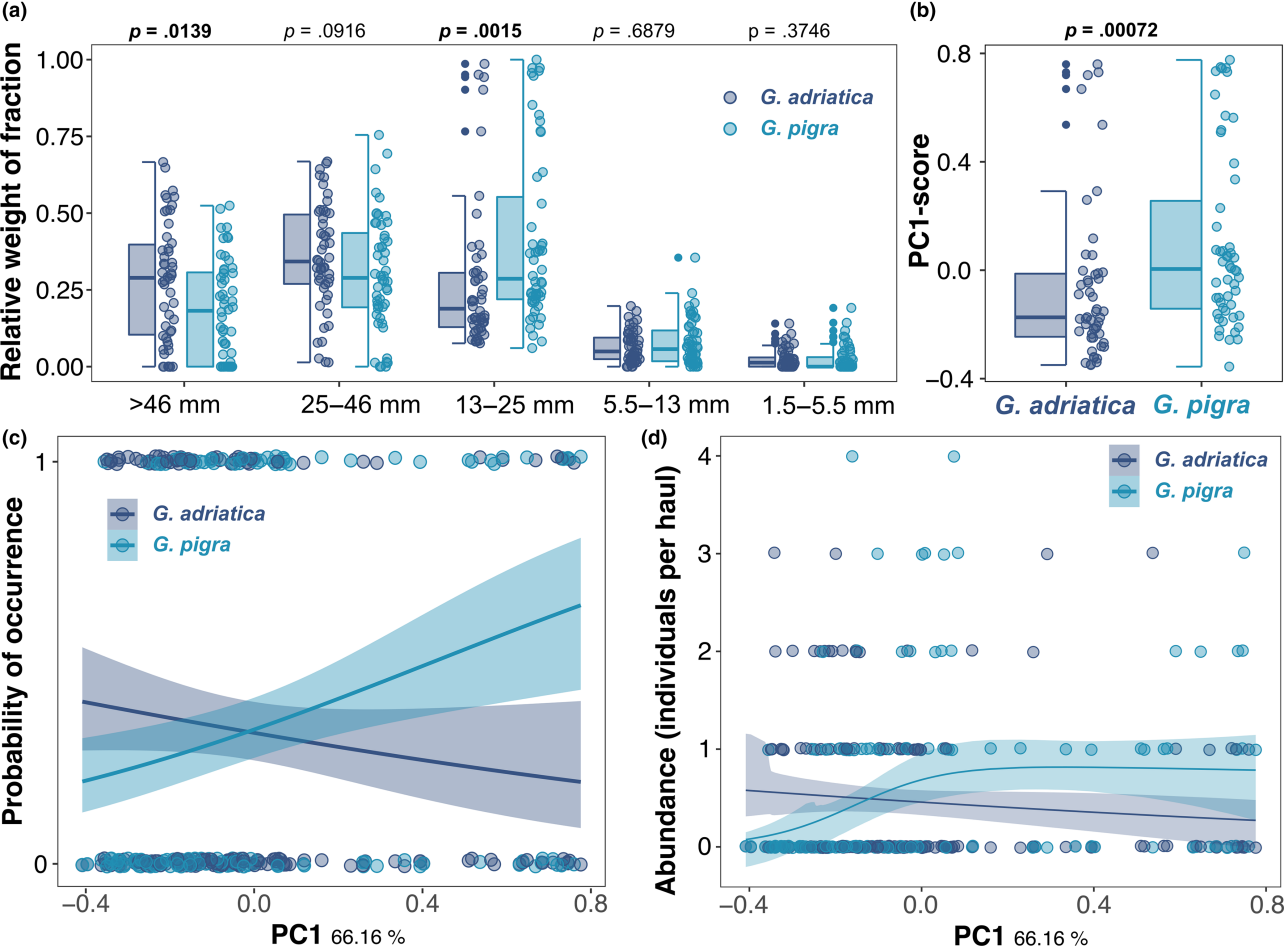
Microhabitat associations of the sympatric *Gouania* species, *G. adriatica* and *G. pigra*. (a) Comparison of relative weight distributions among the five investigated size fractions in hauls that contained either *G. adriatica* (dark blue) or *G. pigra* (light blue). *p*‐values were obtained via Kruskal‐Wallis tests and are in bold font if statistically significant. (b) Differences in the PC1 score of hauls that contained either *G. adriatica* (dark blue) or *G. pigra* (light blue), which is mainly associated with changes in the largest size fraction. (c) Logistic regression models showing the probability of occurrence in hauls based on their PC1‐score for the two species. Lines and confidence bands show the model fit, while jittered dots represent the raw data. The seperate density plot for the absence (0) and presence (1) values for both species can be found in Appendix S10. (d) Abundance of *G. adriatica* and *G. pigra* in hauls based on their PC1‐score. Lines and confidence bands show the model fit from a zero‐inflated Poisson model, while dots represent the raw data.

Across the dataset (and all locations), the two species significantly differed in their association with sediment fractions I (>46 mm) and III (13–25 mm) (Figure 4a), as highlighted by the separation of *G. adriatica* and *G. pigra* on PC1 (Figure 4b; but not for PC2 – see Appendix S7). *Gouania adriatica* was more strongly associated with larger fractions (>46 mm) and *G. pigra* with gravel of intermediate size (13–25 mm). We also observed significant differences between the two species for the sorting index but not for the estimated mean sediment size (Appendix S8). The different microhabitat associations of the two species were supported by significant effects of species affiliation on the presence/absence (*p* = .002; Figure 4c; Appendicies S9 and S10) and abundance of individuals in hauls based on their positioning on PC1 (*p* = .0103; Figure 4d; Appendix S11). Finally, the PERMANOVA revealed that species and the interaction of species with locations correlated significantly with the multivariate distribution of sediment composition in the hauls (*p* = .001 and .011, respectively; Appendix  S4).

**FIGURE 5:**
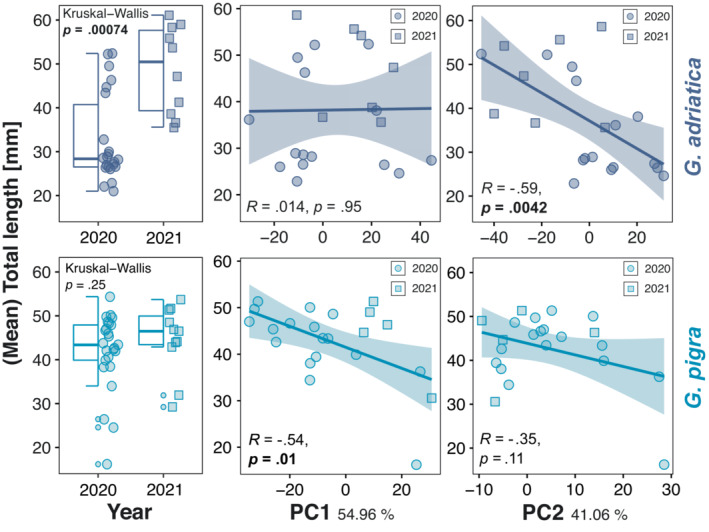
Temporal and ontogenetic determinants of microhabitat occupation for *Gouania adriatica* (top) and *Gouania pigra* (bottom) in Zelenika. The first column indicates changes in total length (TL, in mm) between 2020 and 2021, while the scatterplots display changes in body size across PC1 and PC2 across the 2 years. Each dot in the scatter plots represents a single haul, with shapes indicating the year of collection.

Comparing the samples from two consecutive years at Zelenika, we found significant differences in the total length of *G. adriatica*, but not *G. pigra* (Figure 5). We also observed significant temporal effects on the relative fractions overall (*p* = 0.001, Appendix S12) and particularly, for I as well as V for *G. adriatica* and on the first three fractions (I–III) for *G. pigra* (Appendix S13). Finally, we detected a linear relationship between TL and PC1 for *G. pigra* as well as TL and PC2 for *G. adriatica* (Figure 5).

## 4 DISCUSSION

Intertidal gravel beaches are highly dynamic ecosystems that undergo steady geomorphological changes due to marine erosion and accumulation induced by wave action and long‐shore currents (Duck & da Silva, 2012). This inherent instability may blur species‐specific microhabitat preferences as it hampers the establishment and maintenance of stable ecological boundaries between closely related taxa. Yet, it is not clear how much fine‐scale specificity the denizens of these demanding habitats display, which impairs our understanding of how anthropogenic impacts on gravel beaches may affect their diversity and functioning. Our study shows that sympatric clingfish species in the genus *Gouania* partition their occurrence across gravel beach microhabitats, suggesting species‐specific habitat preferences that may influence their response to natural or anthropogenic alterations of gravel beaches. Our work thus provides important information for shoreline management in the Mediterranean and increases our understanding of eco‐evolutionary dynamics in highly specialized cryptobenthic fishes.

### 4.1 Microhabitat segregation in sympatric Adriatic *Gouania* species

We found evidence for species‐specific microhabitat associations of the two *Gouania* species throughout our study area. Of the 160 investigated samples, only three contained both species and we observed a significant separation of the two species considering their relative sediment distributions. Specifically, grains in the 13–25 mm size range appear to be the preferred microhabitat of *G. pigra*, while the prevalence of *G. adriatica* increased towards the largest observed fraction (>46 mm). Since the grain size of rounded to sub‐rounded shape can serve as a proxy for the size of interstitial space (i.e., larger grains usually provide wider interstitial spaces), these observations are in line with the morphological characteristics of the two species. Slender *G. pigra*, which has a higher body flexibility due to an increased number of vertebrae (Jordan, 1892) and a smaller head, appear to be better adapted to exploit narrower interstitial spaces than the stout *G. adriatica*. An ecological specialization to divergent microhabitats has been previously reported in other closely related interstitial fishes such as gobies (Yamada et al., 2009) and pencil catfishes (Costa et al., 2020) and follows a pattern of adaptive diversification observed in many other cryptobenthic fishes (reviewed by Brandl et al., 2018).

Nonetheless, the observed diverging associations with different gravel environments remained relatively subtle in the present study. This may be due to a variety of reasons, including high variability of grain size in sediment strata that are unoccupied by *Gouania*, strong partitioning of depth zones, or ontogenetic niche overlap. For instance, we found that *G. adriatica* never occurred directly at the waterline and increased in abundance with depth. Yet, *G. adriatica* has previously been recorded above the waterline during spring or neap tides (Hofrichter, 1995; Hofrichter & Patzner, 2000; Wagner et al., 2021), suggesting some temporal variability. Thus, while there is some evidence for depth partitioning – which may coincide with differences in the granulometric composition in the beach body – more targeted sampling under the consideration of fluctuating depths during tidal cycles (despite the rather small tidal ranges in the study area [ca. 35–40 cm]) would be informative. For instance, in this study, we did not differentiate between high and low tides, which should be considered in future studies.

Furthermore, unlike *G. pigra*, which occupies the same gravel size independent of body size, our results suggest an ontogenetic habitat shift in *G. adriatica*. Larvae of *G. adriatica* may recruit into microhabitats occupied by *G. pigra* and shift towards larger‐sized sediments at later life stages. Such developmental microhabitat shifts have been reported in other clingfishes and cryptobenthic fishes and could represent a strategy to mitigate breeding space overlap (Beldade et al., 2006; Gonçalves et al., 2002). Nevertheless, our data clearly suggest non‐random patterns in microhabitat association of *Gouania* species in the Adriatic Sea. Examining whether the same pattern holds true for other sympatric *Gouania* pairs outside the Adriatic Sea (e.g. the slender *G*. *hofrichteri* and stout *G*. *orientalis* from the Eastern Mediterranean Sea; Wagner et al., 2019, 2021) may bolster these findings.

Finally, the role of other factors that lead to niche partitioning among closely related species, such as dietary preferences, remains to be investigated. Generally, food resources (i.e., mainly small crustaceans or snails; see Hofrichter, 1995) are abundant in the interstitial and clingfishes inhabiting primary rocky habitats are considered rather opportunistic feeders (Trkov & Lipej, 2019). Therefore, compared to other cryptobenthic fishes (e.g., Brandl et al., 2020) dietary preferences may contribute less to niche partitioning in *Gouania*. Nonetheless, gut content analyses, ideally through visual assessments and metabarcoding (cf. Casey et al., 2019) may be necessary for unravelling trophodynamics in gravel beaches.

### 4.2 *Gouania* as bioindicators for habitat quality of artificial gravel beaches?

While beaches are relatively rare habitat types in Croatia, accounting for only 5 percent of the total length of the entire eastern Adriatic coastline (Pikelj & Juračić, 2013), they are of increasingly high economic importance. In fact, coastline modification and artificial nourishment of sediments to increase beach surface area are strongly correlated with the growth of tourism over the past decades (Carević, 2020; Carević et al., 2022; Juračić et al., 2009; Pikelj & Juračić, 2013). However, the local and temporal oceanographic conditions are often neglected when designing or managing beach areas. Therefore, especially in anthropogenically affected areas, sediments need to be replenished or re‐nourished annually (Pikelj, Ružić, Ilić, et al., 2018; Speybroeck et al., 2006; Thrush et al., 2003).

The sediment, however, directly influences the biological stability of gravel ecosystems. Throughout our study area, natural sediments of 90% CaCO_3_ (pure limestone and breccias) or 64% to 90% CaCO_3_ (limestones and breccias) were the most common sediment types (Šegina et al., 2021; Velić & Vlahović, 2009). These sediment types are relatively light, soft and prone to erosion and shifts. While this does not represent a problem for natural beaches that are maintained by a complex interplay of biotic and abiotic factors, one prominent artificial nourishment strategy includes waste material from quarries dominated by fine‐grained particles, which are even less durable and more prone to erosion or transportation. This can have negative consequences for the whole interstitial macrofauna, including *Gouania*, which depends on open interstitial space (Carević, 2020; Naqvi & Pullen, 1982; Speybroeck et al., 2006). Indeed, *Gouania* is largely absent at modified or artificial beaches that use waste material (M. Wagner, personal observation). Interestingly, however, we found both *Gouania* species at an artificial gravel beach in Rijeka which consist of highly resistant and durable quartz and metamorphic rocks. This suggests that, although artificial beaches are often unfavourable for beach‐associated biota, artificial beaches composed of grains of appropriate granulometric composition and sourced from natural and more durable, medium‐hard or hard rock types may indeed provide a valuable habitat for *Gouania* and, potentially, a complete and functional interstitial biocenosis.

While substantial efforts have been undertaken to understand and monitor the marine geomorphological dynamics of natural and artificial gravel beaches in the region (Bujak et al., 2021; Lončar et al., 2021; Pikelj, Ružić, Ilić, et al., 2018; Pikelj, Ružić, James, et al., 2018; Ružić et al., 2014, 2019; Tadić et al., 2022), examining the ecological communities of gravel beaches is key to mitigate long term damage to these unique but understudied ecosystems. In this context, our study provides a valuable glimpse into the effects of gravel beach morphology on the structure and function of these ecosystems. Based on their feeding biology (Hofrichter, 1995; Hofrichter & Patzner, 2000), *Gouania* are probably apex predators in the interstitial environment, therefore representing a crucial functional group. Thus, maintaining conditions favourable for *Gouania* species appears advisable. However, a clear understanding of macroinvertebrate biodiversity might be the key to understanding the trophodynamics and ecological functioning of gravel beaches. Therefore, future studies should cast a wider taxonomic net to inform the development of sustainable management strategies.

Currently, the management of beaches in Croatia is performed by regional and local authorities, while appropriate legal frameworks and directives are still to be developed (Pikelj, Ružić, Ilić, et al., 2018). General Croatian policy states that infralittoral gravel beaches are considered part of the protected habitat type sandbanks (i.e., slightly covered by sea water all the time), which means that they are only protected in the ecological network areas designated for this habitat type (Narodne novine 88/2014). Thus, the protection of gravel beach environments underlies only vague legal regulations. Yet, given the high invasiveness of beach nourishment activities (potentially also for surrounding areas; see e.g., Carević, 2020) for the natural world, we propose that environmental impact assessments should precede any anthropogenic alterations in these environments (Staudt et al., 2021).

*Gouania* usually occur in high abundances in suitable habitats and, as apex predators in the gravel beach environment (Hofrichter, 1995; Hofrichter & Patzner, 2000; Wagner et al., 2021), rely on a functional, productive community of smaller fauna to survive. Our study suggests that *Gouania* species associate preferably with specific sedimentary microhabitats, which indicates that severe modifications of the gravel environment, may have detrimental consequences for the two species. Therefore, the genus may serve as an important indicator species for gravel beach management in the Mediterranean area. Given that the method deployed in our study, gravel hauls with buckets, is cheap, relatively easy, and minimally invasive, we suggest that considering *Gouania* in future planning, monitoring, and designing of natural or artificial beaches along Mediterranean coastlines could be a straightforward and pragmatic way to assess the natural condition and state of gravel beach ecosystems. In doing so, we may also make strides towards a better, more holistic understanding of gravel beach ecology and their associated fauna, including secretive, poorly understood cryptobenthic fishes such as *Gouania*.

## AUTHOR CONTRIBUTIONS

**Maximilian Wagner:** Conceptualization (equal); data curation (equal); formal analysis (equal); methodology (equal); visualization (equal); writing – original draft (equal); writing – review and editing (equal). **Čedomir Benac:** Investigation (equal); methodology (equal); validation (equal); writing – original draft (equal). **Maja Pamić:** Conceptualization (equal); investigation (equal); methodology (equal); writing – review and editing (equal). **Sandra Bračun:** Conceptualization (equal); investigation (equal); methodology (equal); writing – review and editing (equal). **Martin Ladner:** Formal analysis (equal); investigation (equal); methodology (equal). **Pia Clarissa Plakolm:** Formal analysis (equal); investigation (equal); methodology (equal). **Stephan Koblmüller:** Resources (equal); supervision (equal); writing – original draft (equal); writing – review and editing (equal). **Hannes Svardal:** Investigation (equal); resources (equal); supervision (equal); validation (equal); writing – original draft (equal); writing – review and editing (equal). **Simon J. Brandl:** Conceptualization (equal); formal analysis (equal); investigation (equal); methodology (equal); supervision (equal); validation (equal); writing – original draft (equal); writing – review and editing (equal).

## CONFLICT OF INTEREST STATEMENT

The authors declare no conflict of interest.

## Supporting information


Appendix S1–S13

## Data Availability

The statistical analyses (R‐scripts) as well as the raw datasets are available and stored at Figshare (https://figshare.com/projects/Gouania_Ecology_paper_raw_data_and_code/155402).
