# Supplementary material for: Microhabitat partitioning between sympatric intertidal fish species highlights the importance of sediment composition in gravel beach conservation
Source: Ecol Evol. 2023 Jul 10;13(7):e10302. doi: 10.1002/ece3.10302 (PMC10333672; doi:10.1002/ece3.10302)
Supplement: Supplementary file 1 — Appendix S1–S13 [file ECE3-13-e10302-s001.docx]

**Supporting information**

**Appendix S1** Supplementary results: In depth characterization of the investigated sampling sites

In the sampling area, Cretaceous carbonate sedimentary rocks (limestones, dolomitic limestones, and carbonate breccias), Paleogene foraminiferal limestones, Paleogene siliciclastic rocks or flysch and Palaeogene-Neogene carbonate breccia were dominant in the terrestrial part of southwestern and eastern Istria peninsula and Krk Island, and on the northern coast of Rijeka bay. The allochthonous gravel found at the beach in Rijeka contained grains of quartz and metamorphic rocks that do not occur in the wider Rijeka area, but likely originated from alluvial deposits of the Drava River in northern Croatia.

Different patterns in the overall relative contribution of each of the five gravel fractions were observed from different sampling sites (Figure 2b). The three beaches from the southern Istrian peninsular, Muzil, Zelenika and Cava, predominantly show a decrease in the relative weight from the largest to the smallest fraction. A distribution peaking in the second largest fraction II (25 – 46 mm) was observed at the site around the island of Krk (Glatovok, Zala and Surbova). At Sv. Marina the sediment amount increased from fraction I to III and subsequently decreased. Rijeka displayed a special case, where gravels of fraction III (13 – 25 mm) dominated throughout the beach and other fractions only made up a minimal part of the total sediment. On all investigated beaches, the smallest fraction V (1.5 – 5.5 mm), contributed the least to the total sediment amount in the hauls, except for the site Rijeka where the largest fraction was largely absent.

**(1) Muzil, Muzilj cove**

Muzilj cove is located on the west coast of the Muzil peninsula in southwestern Istria (Figure 2, location map). The beach is exposed to wind and waves from the southwestern direction. The sides of the cove are formed by well-layered Lower Cretaceous limestone. The beach body is about 20 m long and 25 m wide. A 10 m wide part of this body is below the M.S.L. The sea bottom of the cove is partially covered with sand and outcrops of limestone bedrock can be visible (Figure 2).

This beach originated through long-term marine erosion of limestone rock mass. The gravel and pebbles of the beach body are composed of the surrounding limestone rock. The predominant grain size is 25 to 70 mm. Its sediments are rounded to sub-rounded and have an equant and bladed shape. Fragments of fine to medium gravel (2-10 mm) make up less than 10% of the total volume of the sediment body.

**(2) Zelenika, Stoja cove**

The studied beach is located in the northeastern part of Stoja cove, in the southwestern part of the Istrian peninsula (Figure 2, location map). This cove is partially exposed to wind and waves from the southern direction and was formed by well-layered Lower Cretaceous limestone. The beach body is approximately 80m long and 15 to 20m wide. A 10 to 15 m wide part of the body is located below the M.S.L. The sea bottom of the cove is partially covered with sand and outcrops of carbonate bedrock can be visible (Figure 2). This beach was formed through long-term marine erosion of limestone rock mass. The gravel and pebbles of the beach body are composed of the surrounding limestone rock. The grain size of the beach is 10 to 70 mm. These grains are rounded to sub-rounded and have equant and discoidal shapes.

**(3) Cava, Kaval cove**

Kaval cove is located, on the east coast of the Istrian peninsula (Figure 2, location map). The cove is exposed to wind and waves from the northeast and east, and partly from the south. The coastline is composed of Upper Cretaceous rudist limestones. The beach body is 70 m long and 30 m wide. A 15 m wide of this body is below the M.S.L. The sea bottom of the cove is covered with sand, and outcrops of carbonate bedrock are visible (Figure 2). The sediment of the beach has a natural origin from long-term marine erosion of the surrounding limestone rock masses. The dominant grain size is 14 to 50 mm. These grains are rounded to sub-rounded and have equant and spherical shapes. Fragments of coarse sand to fine gravel (1-3 mm) make up less than 10 % of the total volume of the sediment body.

**(4) Sv. Marina, Guboka cove**

The studied beach is located south of St. Marina Bay on the eastern side of the Istrian peninsula (Figure 2). Guboka cove is exposed to wind and waves from northeastern and eastern directions and is composed of Upper Cretaceous rudist limestones. The beach body is 90 m long and 30 m wide. A 17 m wide part of this body is below the M.S.L. The sea bottom of the cove is covered with sand and outcrops of carbonate bedrock are visible (Figure 2). The sediment of the beach arose from long-term marine erosion of the surrounding limestone rock mass and the transport of fragments by the periodic torrent from the upstream part of the slope. The grain size of the beach body is 5 to 35 mm. These grains are rounded to sub-rounded and have equant, spherical and discoidal shapes.

**(5) Rijeka, Pećine**

The studied beach is located southeast of the mouth of the Rječina River near the center of Rijeka (Figure 2). This small cove is exposed to wind and waves from western and southwestern directions and is composed of Upper Cretaceous limestones and dolomitic limestones. The beach body is 20 m long and 13 m wide. A 10 m wide part of this body is below the M.S.L. Older sediment of the beach is of natural origin, formed via long-term marine erosion of carbonate rock mass. Thus, the deeper zone of the sea bottom is partially covered by pebble and cobble (Figure 2). However, the beach was artificially nourished with gravelly sediment, approximately ten years ago. This allochthonous sediment probably originated from the alluvial deposits of the Drava River in northern Croatia. This sediment contains grains of quartz and metamorphic rocks that do not occur in the wider Rijeka area.

For this reason, allochthonous sediment predominates in the present beach body, but individual grains from the older carbonate rocks may also be found. The grain size ranges from 16 to 35 mm. These grains are rounded to sub-rounded and have an equant, spherical and discoidal shape.

**(6) Glavotok, Komoštrin cove**

The studied beach is located south of the settlement of Glavotok on the west coast of Krk Island (Figure 2). This small cove is exposed to wind and waves from western and southwestern directions and was formed by Upper Cretaceous rudist limestones, dolomitic limestones and also Palaeogene foraminiferal limestone. The beach body is 30 m long and 10 m wide. A 5 m wide part of this beach body is below the M.S.L. The sea bottom of the cove is partially covered with sand and outcrops of carbonate bedrock are visible (Figure 2). The formation of this beach is a consequence of long-term marine erosion of the surrounding carbonate rock mass, thus reflecting the natural substratum of the cove. The predominant grain size is 10 to 70 mm. These grains are rounded to sub-rounded and have an equant and spherical shape. Fragments of coarse sand to fine gravel (1-5 mm) make up less than 10 % of the total volume of the sediment body.

**(7) Zala, Zala cove**

Zala cove is located on the southeastern coast of Krk Island (Figure 2). The beach is exposed to the wind and waves from the southern direction. The sides of Zala cove are formed from Palaeogene foraminiferal limestones. The beach body is about 100 m long and 30 to 40 m wide. A 20 to 30 m wide part of it is below the M.S.L. The bottom of the cove is covered with sand and outcrops of carbonate bedrock are visible (Figure 2). The sediment of the beach arose from long-term marine erosion of the surrounding foraminiferal limestones and gravitational transport of fragments from Upper Cretaceous limestones visible on the surface of outcrops above the cove. Some red grains from Upper Cretaceous breccias are also found in the sediment body. The grain size is 4 to 40 mm. The grains are rounded to sub-rounded and have a spherical, equant and discoidal shape.

**(8) Surbova, Surbova cove**

Surbova cove is located on the southeastern coast of Krk Island (Figure 2). The beach is exposed to the wind and waves from the southwestern direction. The sides of Surbova cove are formed from Palaeogene foraminiferal limestones. The beach body is about 70 m long and 25 to 35 m wide. A 15 to 25 m wide part of this body is located below the M.S.L. The bottom of the cove is covered with sand and outcrops of carbonate bedrock are also visible (Figure 2). The sediment of the beach is a consequence of long-term marine erosion of surrounding foraminiferal limestones and gravitational transport of fragments from Upper Cretaceous limestones visible on the surface of outcrops above the cove. Some red grains from Upper Cretaceous breccia are also found in sediment body. The grain size is from 5 to 50 mm in the beach body. The grains are rounded to sub-rounded and have a spherical, equant and discoidal shape.


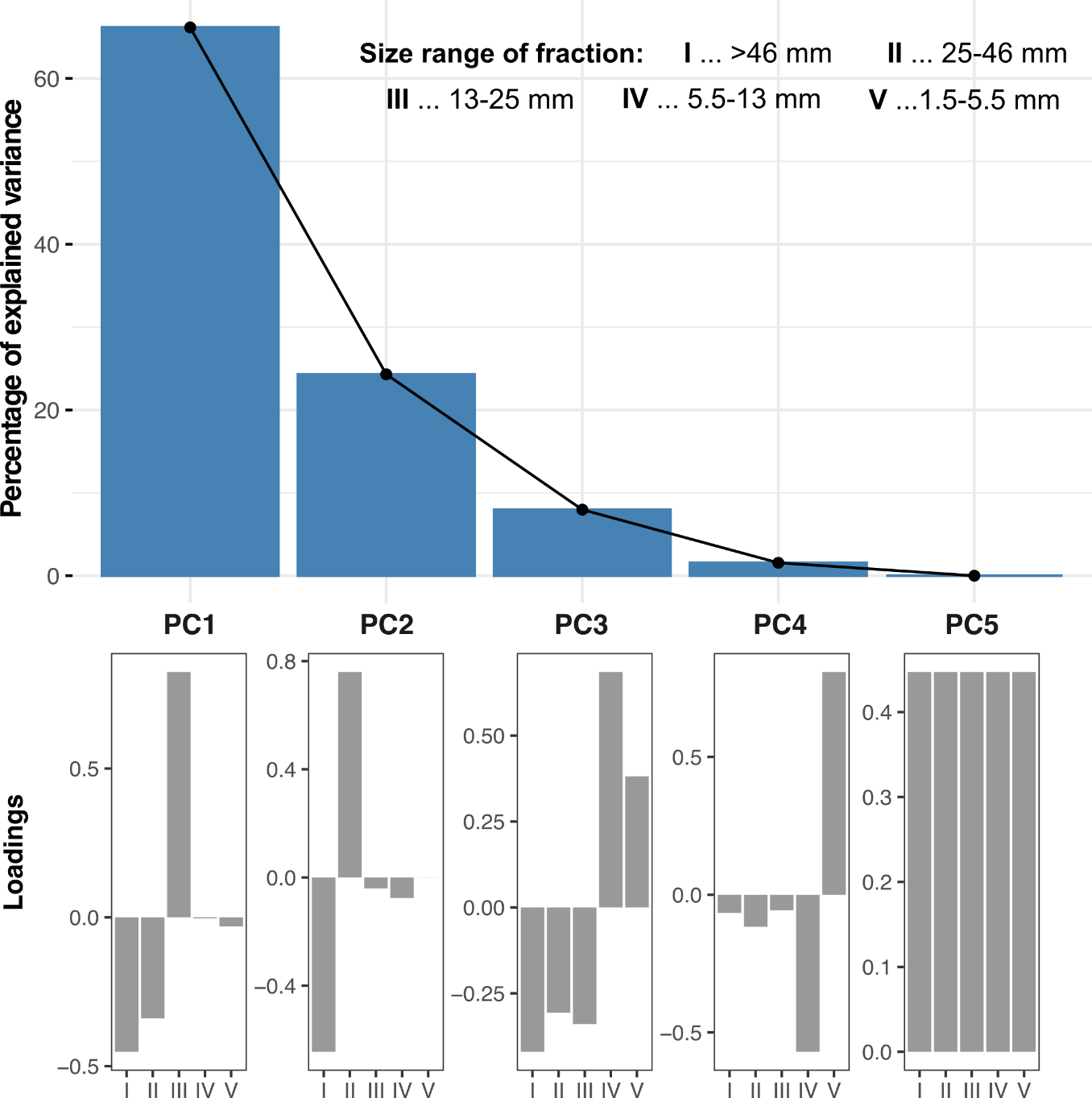


**Appendix S2** Scree plot and loadings for the Principal components analyses (PCA) based on the whole dataset shown in Figure 2 and 3.


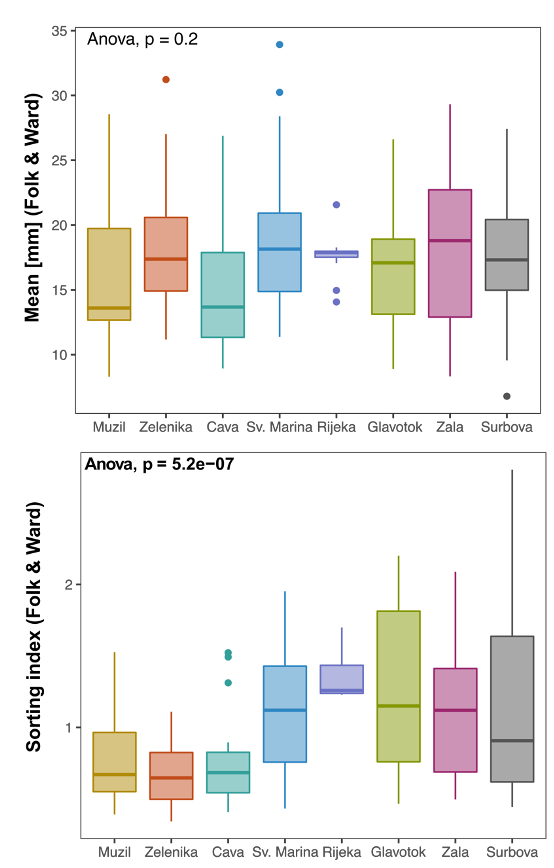

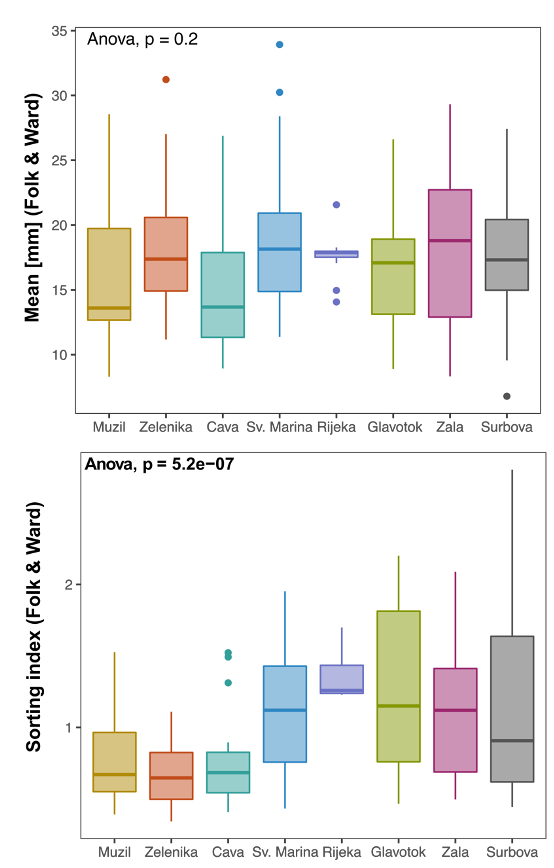


**Appendix S3** Comparison of the estimated mean sediment size (mm) and sorting index, calculated following Folk & Ward (1957) for the different locations.

**Appendix S4** PERMANOVA results from the multivariate analyses of hauls based on their sedimentary composition

|  | Df | SumsOfSqs | MeanSqs | F.Model | R2 | Pr(>F) |
| --- | --- | --- | --- | --- | --- | --- |
| Species | 1 | 0.4832 | 0.48324 | 10.6736 | 0.04702 | **0.001** |
| Location | 7 | 5.2161 | 0.74516 | 16.4588 | 0.50752 | **0.001** |
| Species:Location | 7 | 0.6846 | 0.0978 | 2.1603 | 0.06661 | **0.013** |
| Residuals | 86 | 3.8936 | 0.04527 |  | 0.37884 |  |
| Total | 101 | 10.2776 |  |  | 1.00000 |  |

**
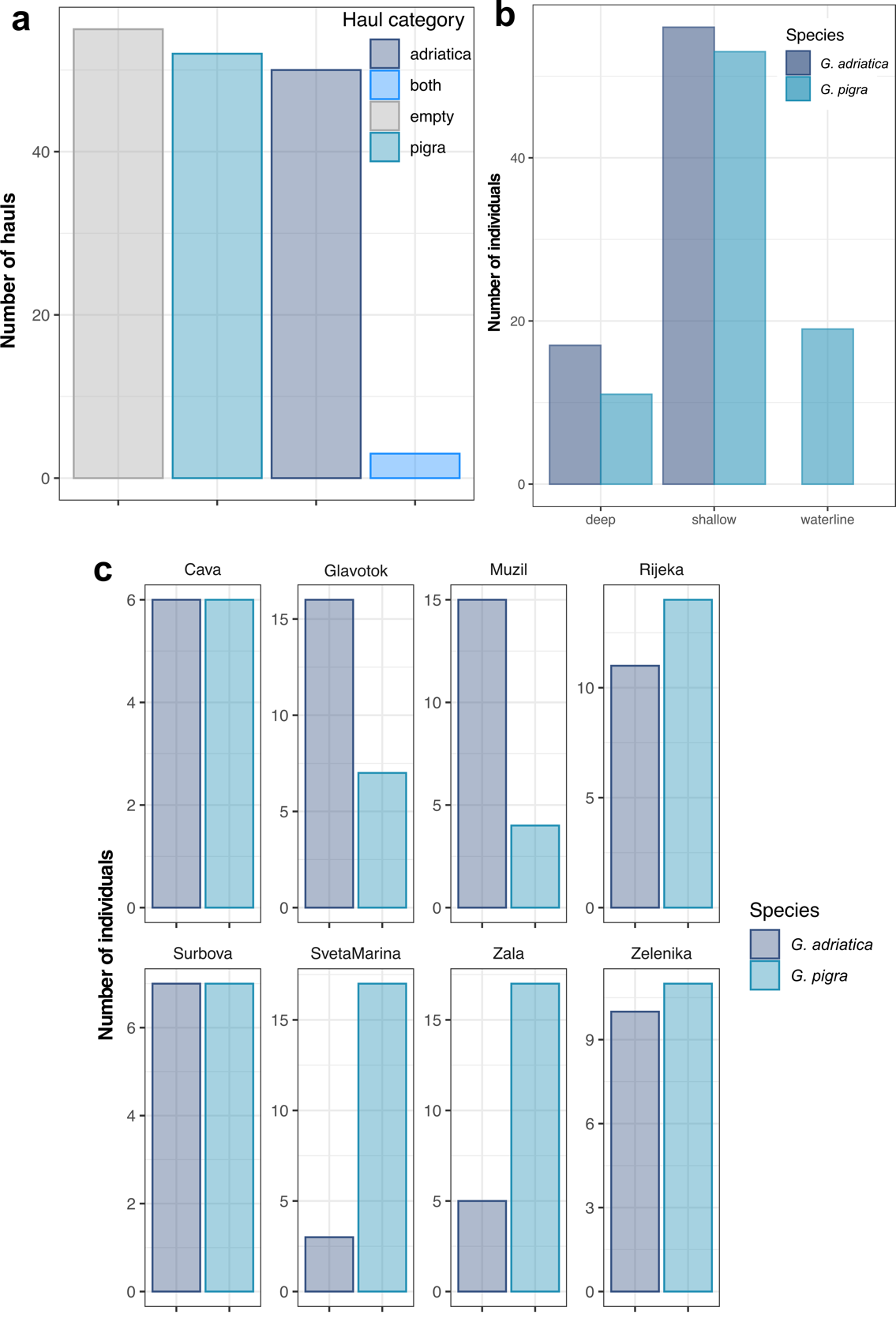
**

**Appendix S5** Sampling overview. (a) Number of hauls where both, none or one of each species, *G. adriatica* or *G. pigra* were found. b) and c) Number of individuals from the two species at different depths (b) and locations (c).


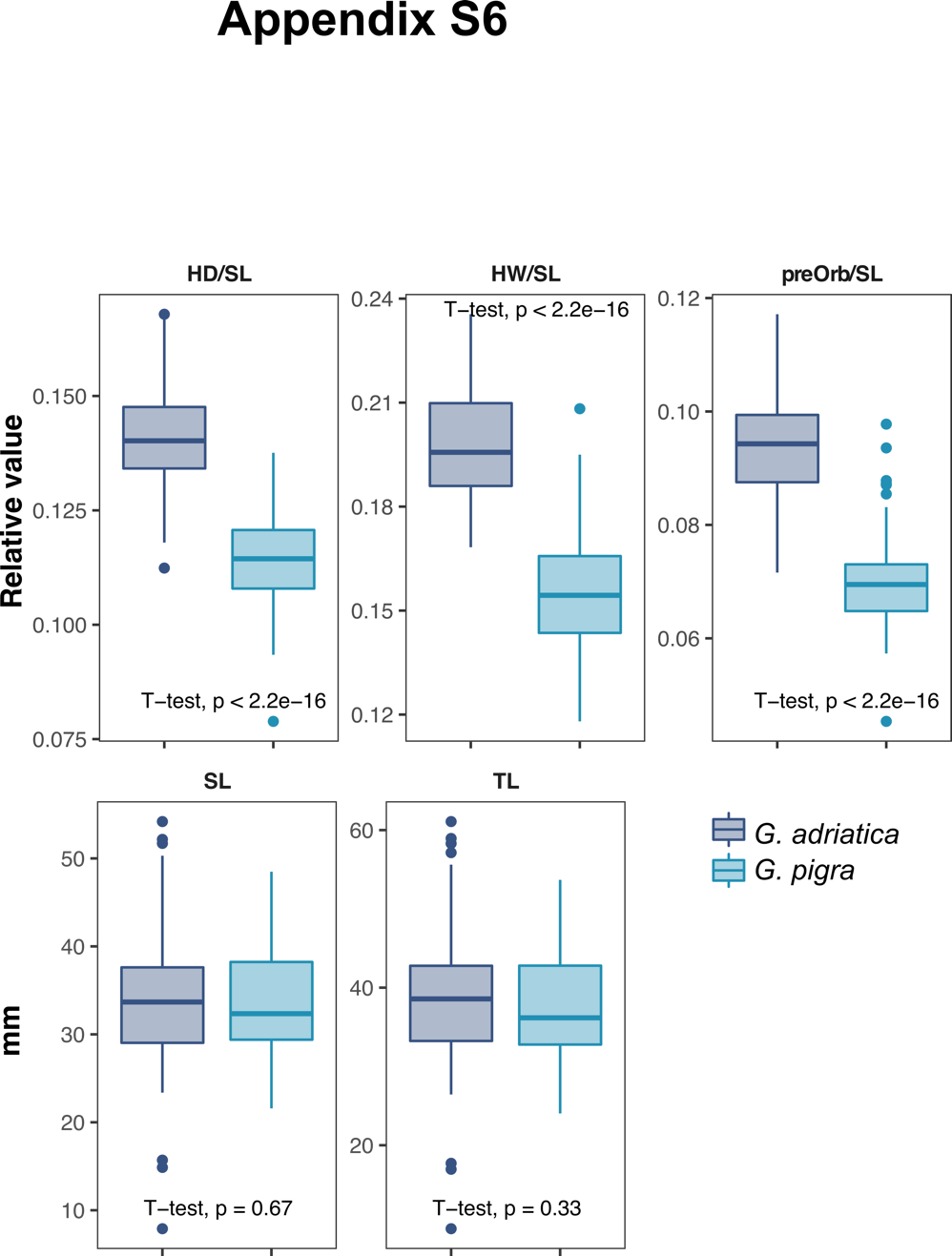


**Appendix S6** Relative measurements relative to standard length (SL, first row) as well as SL and TL measured in mm. Abbreviations: SL: standard length, TL: total length, preOrb: preorbital distance, HD: head depth, HW: head width.


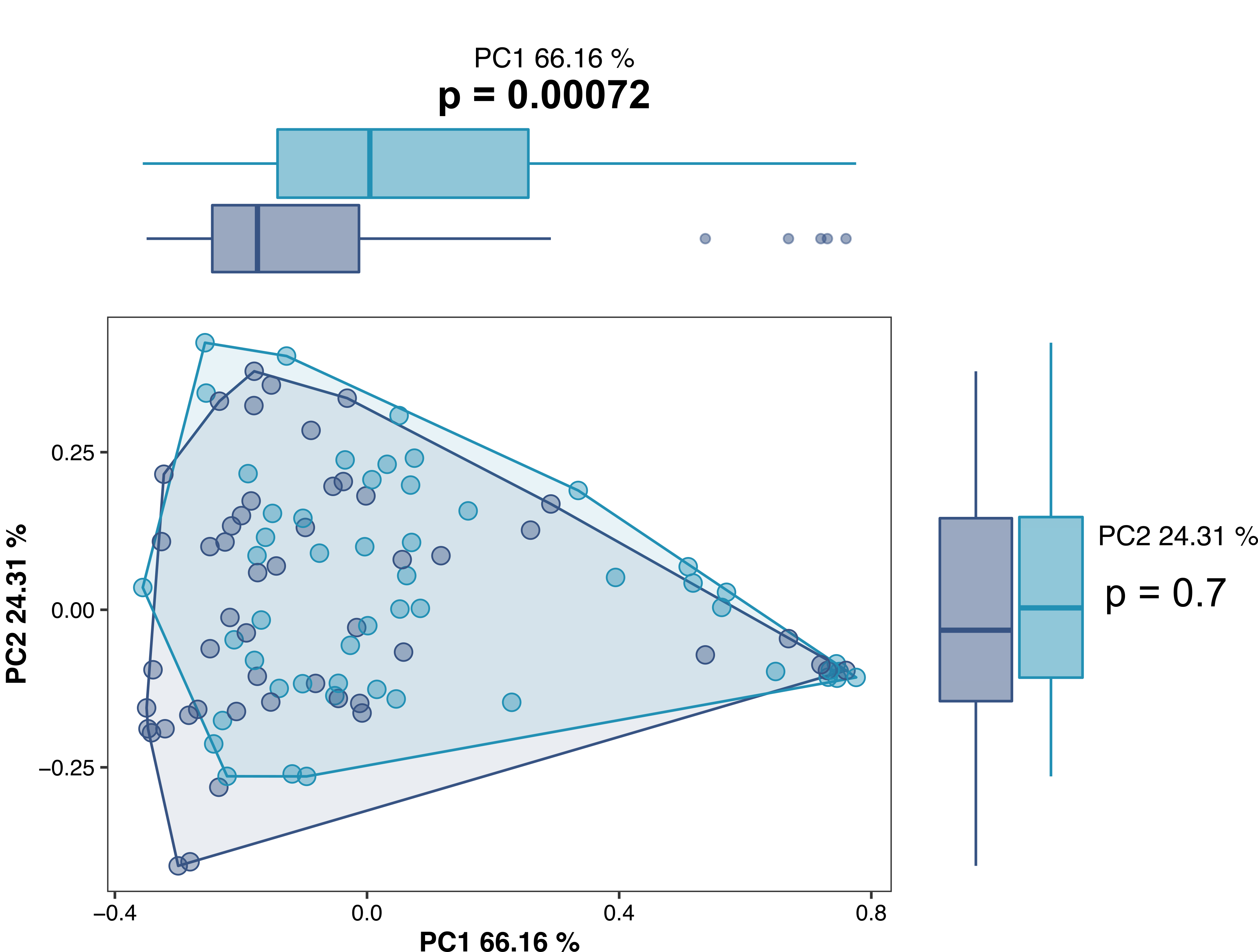


**Appendix S7** Species specific distributions across hauls in two-dimensional space as yielded by the PCA based on the whole dataset shown in Figure 2 and 3.

**
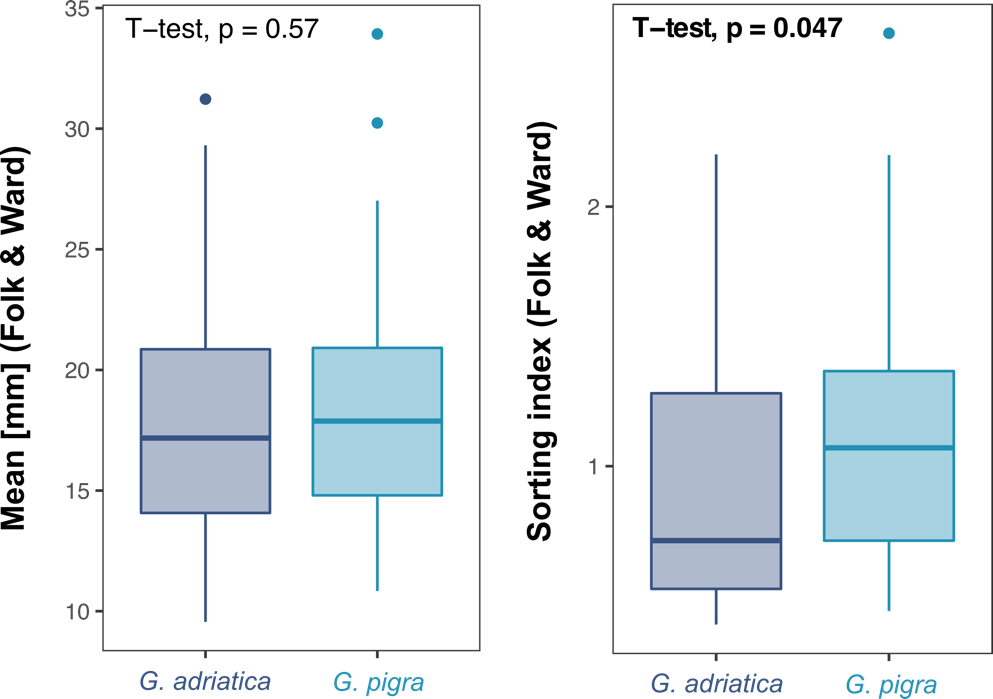
**

**Appendix S8** Comparison of the mean sediment size (mm) and sorting index for the two species.

**Appendix S9** Model coefficients obtained for the logistic regression shown in Figure 4c.

|  | Estimate | Std. Error | z-value | Pr(>\|z\|) |
| --- | --- | --- | --- | --- |
| (Intercept) | -0.74341 | 0.17084 | -4.351 | **1.35E-05** |
| Spcecies | 0.03766 | 0.24347 | 0.155 | 0.87706 |
| PC1 | -0.82648 | 0.58599 | -1.41 | 0.15842 |
| Species:PC1 | 2.47525 | 0.7963 | 3.108 | **0.00188** |


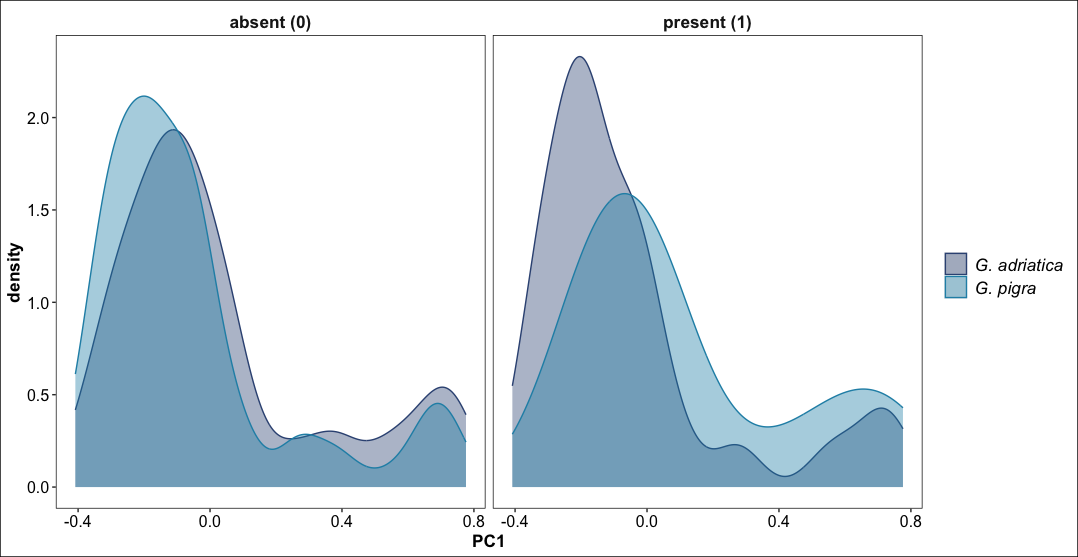


**Appendix S10** Density plot for absence/presence data (Figure 4c).

**Appendix S11** Zero-inflation model coefficients (binomial with logit link) from the model shown in Figure 4d.

|  | Estimate | Std. Error | z-value | Pr(>\|z\|) |
| --- | --- | --- | --- | --- |
| (Intercept) | -0.4797 | 0.4761 | -1.008 | 0.3137 |
| PC1 | 0.8370 | 12.374 | 0.676 | 0.4988 |
| Species | -0.9212 | 10.239 | -0.900 | 0.3683 |
| PC1: Species | -101.340 | 39.499 | -2.566 | **0.0103** |

**Appendix S12** Results obtained from PERMANOVA from the temporal comparison.

|  | Df | SumsOfSqs | MeanSqs | F.Model | R2 | Pr(>F) |
| --- | --- | --- | --- | --- | --- | --- |
| Species | 1 | 0.10911 | 0.10911 | 2.4787 | 0.04839 | 0.087 |
| Year | 1 | 0.32063 | 0.32063 | 7.2836 | 0.14220 | **0.001** |
| Species:Year | 1 | 0.06424 | 0.06424 | 1.4592 | 0.02849 | 0.227 |
| Residuals | 40 | 1.76083 | 0.04402 |  | 0.78092 |  |
| Total | 43 | 2.25481 |  |  | 1.00000 |  |

**
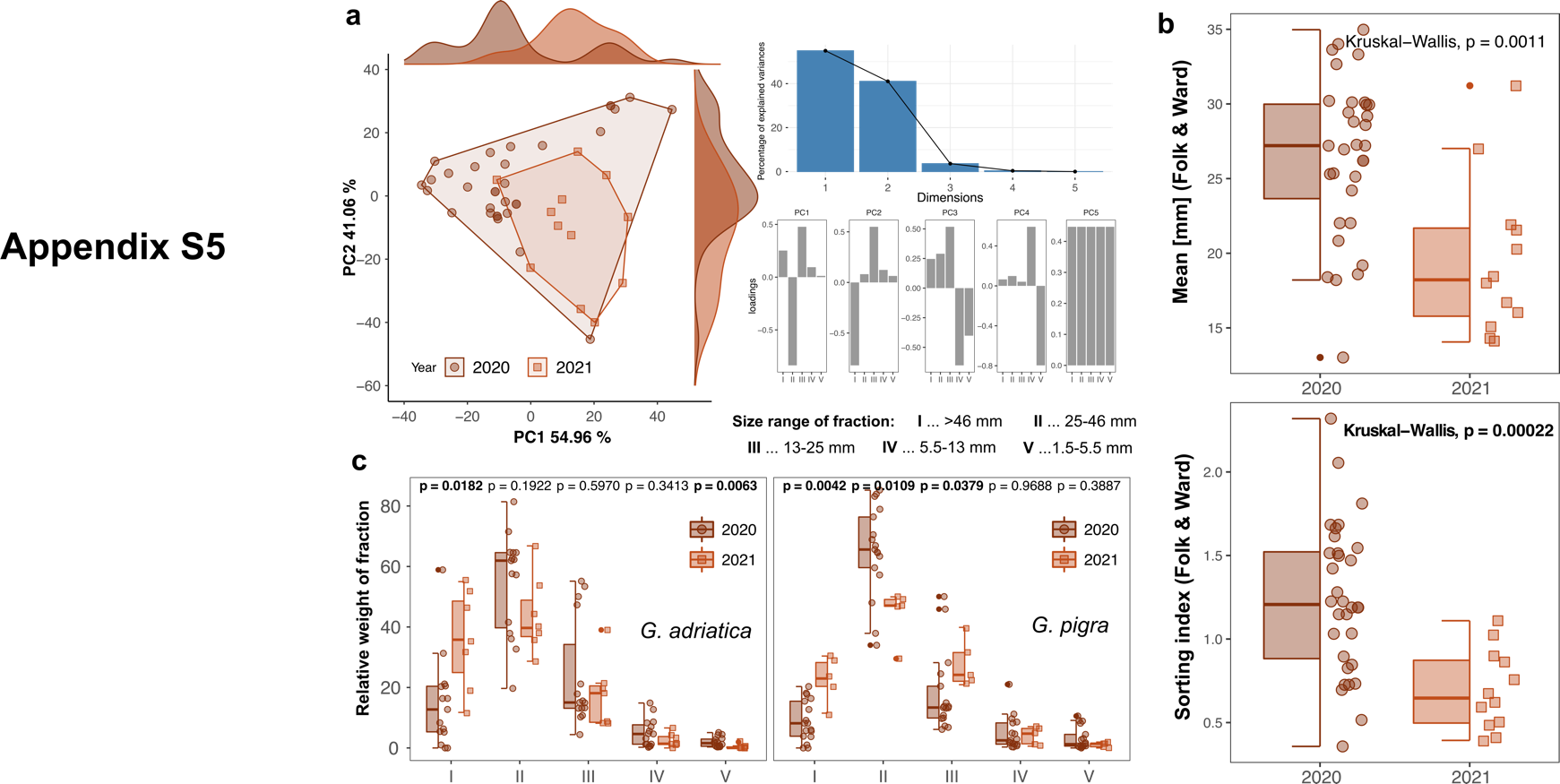
**

**Appendix S13** Temporal geomorphological changes at the beach in Zelenika. (a Principal components analysis (PCA) including the screeplot and loadings). (b) Comparison of the two geological parameters Mean (mm) and Sorting index calculated after the formulas of Folk & Ward (1957) for the different locations. (c) Relative geomorphological changes at the beach Zelenika for both species and sampling times.
